# The surgical outcomes of fixing ipsilateral femoral neck and shaft fractures: single versus double implants fixation

**DOI:** 10.1007/s00590-022-03312-0

**Published:** 2022-07-04

**Authors:** Yahya Alborno, Abdullah Abunimer, Yousef Abuodeh, Motasem Salameh, Hammam Kayali, Ghalib Ahmed

**Affiliations:** 1 Department of Orthopedic Surgery, Hamad General Hospital, Doha, Qatar; 2 Miller School of Medicine, University of Miami Hospital-Jackson Memorial Hospital, Miami, USA; 3 Orthopedic Surgery Department, Brown University, Providence, RI USA

**Keywords:** Femur, Fracture fixation, Ipsilateral, Neck, Union

## Abstract

**Background:**

Combined ipsilateral femoral neck and shaft fractures are rare and present a challenging management dilemma. This study aims to assess the outcome of concomitant fixation of the ipsilateral femoral neck and shaft fracture using single versus dual surgical implants.

**Methods:**

A single-center retrospective analysis of patients who underwent fixation of ipsilateral femoral neck and shaft fractures was performed over a 13-year period. Different techniques were employed to fix the complex injury. Both the reduction and the union time were assessed radiographically.

**Results:**

A total of 36 patients with ipsilateral femoral neck and shaft fractures were retrospectively identified and included in the study. Twenty-four patients (66.6%) were managed with a single cephalomedullary nail, while the remaining cases were treated with two devices. All cases were operated on within an average of 3.7 ± 7.1 days. Eight patients (22.2%) developed postoperative complications. The average follow-up period was 7.3 ± 6.8 months. Although there was no statistically significant difference between the two groups, the femoral neck fractures showed shorter union time in patients treated with one implant compared to patients treated with two implants (3.0 ± 2.3 months vs. 4.2 ± 2.6 months). Another observation was that higher percentages of implant removal/failure and malunion/nonunion were seen in patients who had one implant compared to the two implants group (12.5% vs. 8.3%).

**Conclusion:**

Early surgical fixation of both fractures is associated with good outcome results. No difference in outcome was observed between both groups.

**Supplementary Information:**

The online version contains supplementary material available at 10.1007/s00590-022-03312-0.

## Introduction

Combined ipsilateral femoral neck and shaft fractures are rare and present a challenging management dilemma. The incidence ranges between three and ten percent [1] and usually occurs due to high energy trauma. The diagnosis of associated neck fracture can be missed easily or delayed, in up to one-third of the cases [2, 3], and therefore, it is essential to perform a thorough evaluation of polytrauma patients with diaphyseal femoral fracture[4–6]. Currently with the improvement of trauma care and increased survival rate of polytrauma patients, the incidence is more likely to increase.

The mechanism of injury usually involves axial compression against the acetabulum, with concomitant adduction or abduction of the hip [7]. The femoral shaft fracture usually is more comminuted as it absorbs most of the energy, while the neck usually sustains less displacement [2]. Regarding the ideal management strategy, there has been a lot of controversy about the optimal fixation technique of these combined injuries. The increased risk of avascular necrosis with delayed fixation of the femoral neck has driven surgeons to prioritize early anatomical fixation of the neck fracture [8]. Whether this was achieved using a single versus dual implants is still controversial. This study aimed to assess the outcome of concomitant fixation of the ipsilateral femoral neck and shaft fracture using single versus dual surgical implants.

## Methods

### Data acquisition

This study has been reported in line with strengthening the reporting of observational studies in epidemiology (STROBE) guidelines [9]. After receiving institutional review board approval with a waiver of consent, during the period from January 2008 and August 2020, all cases who underwent femoral shaft fixation in a single-level 1 trauma center were reviewed. Out of 1861 cases, 36 cases of concomitant ipsilateral femoral neck and shaft fractures were identified and included in the study. The inclusion criteria were patients > 17 who underwent surgical fixation of ipsilateral femoral shaft and neck fractures with at least 6 months of follow-up.

Demographic and surgical characteristics were reviewed and included patients’ age, gender, mechanism of injury, location of femoral neck fracture (basicervical or transcervical), displacement of femoral neck fracture classified as nondisplaced (Garden classification grade one and two) or displaced (Garden classification grade three and four), location of femoral shaft fracture (proximal, midshaft or distal), other associated injuries, time to surgery, surgical positioning, methods of reduction, number of implants used, postoperative complications, postoperative union time, implant removal/failure, malunion/nonunion and duration of follow-up. The decision whether to fix the fractures using one or two implants was made by the primary surgeon. Several factors affected the selection process, including the patient’s general condition, the amount of displacement of a neck fracture and the surgeon’s preference.

The primary outcome was fracture radiographic union defined by evidence of bridging callus on three out of four cortices on anteroposterior and lateral radiographs. The secondary outcome is the accuracy of reduction which was evaluated for both femoral neck and shaft fractures. The reduction of femoral neck fracture was assessed radiographically based on two parameters according to Haidukewych et al. [10]: the degree of residual angulation and the amount of displacement (cortex apposition regardless of direction). Excellent reduction was defined as < two mm of displacement and < five degrees of angulation in any plane, good as two to five mm displacement and/or five to ten degrees of angulation, fair as > five to ten mm of displacement and/or > 10–20° of angulation and poor as > 10 mm of displacement and/or > 20° of angulation. Similarly, femoral shaft malreduction was defined as > 5° of angulation in the coronal plane or > 10° of angulation in the sagittal plane [11]. In addition, implant failure was defined as femoral neck screws cut out, breakage of nail or breakage of proximal or distal locking screws. All radiographs were reviewed using the picture archiving and communication system (PACS) by one orthopedic surgery resident and a trauma fellowship trained orthopedic surgeon, and any conflict was resolved by the senior author (G.A.)

### Statistical analysis

Continuous variables were reported as a mean ± SD and categorical variables as frequency (%). A p-value of ≤ 0.05 was considered statistically significant. Comparison between one implant group and two implants group was done using an independent t test. The rate of complications and union rate between one implant versus two implants were analyzed using Mann–Whitney *U* test. Data were analyzed using SPSS version 11.0 statistic software package.

## Results

A total of 36 cases of ipsilateral femoral neck and shaft fractures were retrospectively identified and included in the study (Table 1). In our sample, the mean age was 37.5 ± 11.1 years, with males representing most of the cases (83.3%). The mechanism of injury was fall from height in 30 cases (83.3%), while 6 cases (16.7%) had fractures secondary to motor vehicle collisions. Among the studied cases, one patient had bilateral neck and shaft fractures, while the rest sustained unilateral injuries. Twenty-nine patients (80.6%) sustained other associated musculoskeletal, head, chest and abdominal injuries. All cases were operated on within an average of 3.7 ± 7.1 days.

**Table 1:** Demographic and surgical characteristics of 36 patients with ipsilateral femoral neck and shaft fractures

Variable	Measurement
Age (years), mean ± SD	37.5 ± 11.1
Male gender (% total)	30 (83.3%)
Mechanism of injury (% total)	
Fall from height	30 (83.3%)
Motor vehicle collision	6 (16.7%)
Associated injuries (% total)	29 (80.6%)
Time to surgery (days), mean ± SD	3.7 ± 7.1
Surgical positioning (% total)	
Supine	25 (69.4%)
Lateral	11 (30.6%)
Reduction method (% total)	
Closed	32 (88.9%)
Open	4 (11.1%)
Number of implants (% total)	
One	24 (66.7%)
Two	12 (33.3%)
Postoperative complications (% total)	8 (22.2%)
Union time (months), mean ± SD	
Femoral neck	3.4 ± 2.4
Femoral shaft	5.5 ± 4.2
Implant removal/failure (% total)	4 (11.1%)
Malunion/nonunion (% total)	4 (11.1%)
Duration of follow-up (months), mean ± SD	7.3 ± 6.8

The fracture characteristics of our cohort are summarized in Table 2. Femoral neck fractures were basicervical in 17 cases (47.2%) and transcervical in 19 cases (52.8%). Displacement was found in 14 cases of neck fractures (38.9%), while the remaining 22 (61.1%) were either Garden’s grade I or II. The mean Pauwels’s angle was 65.8 ± 14.8 degrees. Regarding the shaft fractures, they were located in the midshaft in the majority of the cases (77.8%). The average neck-shaft angle was 128.6 ± 5.2 degrees.

**Table 2:** Fracture characteristics of 36 patients with ipsilateral femoral neck and shaft fractures

Variable	Measurement
Location of femoral neck fracture (% total)	
Basicervical	17 (47.2%)
Transcervical	19 (52.8%)
Displacement of femoral neck fracture (% total)	
Non-displaced	22 (61.1%)
Displaced	14 (38.9%)
Femoral neck Pauwels’s angle (degrees), mean ± SD	65.8 ± 14.8
Location of femoral shaft fracture (% total)	
Proximal	4 (11.1%)
Midshaft	28 (77.8%)
Distal	4 (11.1%)
Neck-shaft angle (degrees), mean ± SD	128.6 ± 5.2

**Fig. 1:**
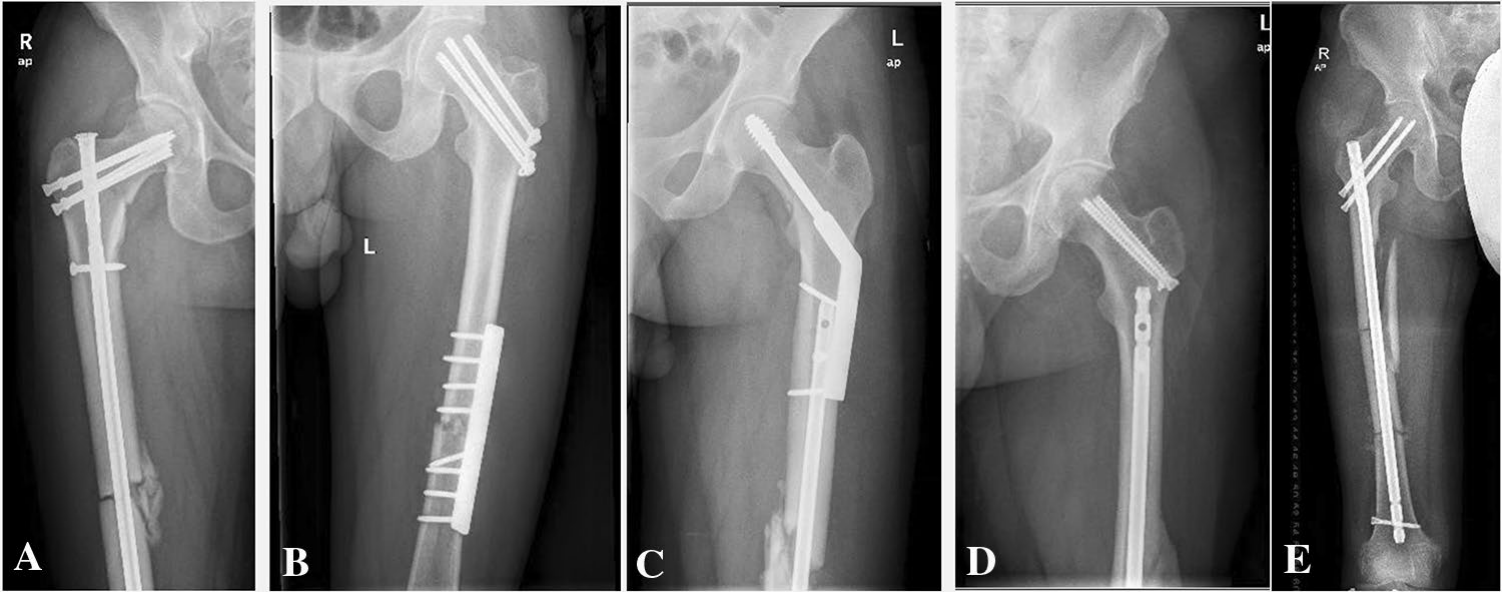
Different surgical techniques for the management of ipsilateral femoral neck and shaft fractures. **A** Antegrade femoral nailing with cancellous screws placed around the nail for the neck fracture. **B** Plate fixation of the diaphyseal fracture with cancellous screw. **C** Retrograde nailing with dynamic hip screw. **D** Retrograde nailing with cannulated screws. **E** Cephalomedullary nail

Different techniques were used to fix the concomitant ipsilateral femoral neck and shaft fractures (Fig. 1). Twenty-four patients (66.6%) were managed with a single cephalomedullary nail (CMN). The remaining cases were treated with two devices (seven patients received retrograde nailing and cannulated screws, two patients had antegrade nailing and cannulated screws, two patients had a sliding hip screw and retrograde nailing, and one patient had plate fixation and cannulated screws). Although there was no statistically significant difference between the two groups, all patients fixed with two devices had either good or excellent femoral neck reduction, while good or excellent reduction of neck fracture was noted in 84.7% of patients managed with one device. Femoral shaft malreduction was observed in three patients treated by one implant method and only one patient from the two devices group.

Upon comparing the patients who were fixed with one implant (*N* = 24) versus two implants (*N* = 12), none of our prognostic factors showed statistical significance (Table 3). However, the femoral neck fractures showed a shorter union time in patients treated with one implant compared to patients treated with two implants (3.0 ± 2.3 months vs. 4.2 ± 2.6 months). Another observation was that higher percentages of implant removal/failure and malunion/nonunion were seen in patients who had one implant compared to the two implants group (12.5% vs. 8.3%).

**Table 3:** Comparison of one versus two surgical implants for the management of ipsilateral femoral neck and shaft fractures

Variable	One implant (*N* = 24)	Two implants (*N* = 12)	*p* value
Age (years), mean ± SD	38.1 ± 12.4	36.3 ± 8.1	0.6
Male gender (% total)	19 (79.2%)	11 (91.7%)	0.6
Postoperative complications (% total)	5 (20.8%)	3 (25.0%)	0.9
Union time (months), mean ± SD			
Femoral neck	3.0 ± 2.3	4.2 ± 2.6	0.2
Femoral shaft	5.5 ± 5.0	5.3 ± 2.3	0.9
Implant removal/failure (% total)	3 (12.5%)	1 (8.3%)	0.6
Malunion/nonunion (% total)	3 (12.5%)	1 (8.3%)	0.7
Displacement of femoral neck fracture (% total)			
Non-displaced	12 (50.0%)	10 (83.3%)	
Displaced	12 (50.0%)	2 (16.7%)	0.3
Location of femoral shaft fracture (% total)			
Proximal	3 (12.5%)	1 (8.3%)	
Midshaft	19 (79.2%)	9 (75.0%)	0.6
Distal	2 (8.3%)	2 (16.7%)	

Eight patients (22.2%) developed postoperative complications. In the single device group, one case developed wound infection, three cases developed nonunion, and three cases developed malunion. Only one case from the dual devices group developed malunion. The average follow-up period was 7.3 ± 6.8 months.

## Discussion

In this retrospective analysis of 36 cases of ipsilateral femoral neck and shaft fractures, no difference in the radiological outcomes and complications for patients treated with single versus dual construct was reported. Although higher rate of excellent reduction of both the femoral neck and shaft was found in the two device group, this did not reach statistical significant. On the other hand, single construct fixation showed and average 1.2 months faster time to union when compared to dual construct fixation with no statistical significance. Our results were consistent with the available literature with no solid conclusions can be made on the optimal construct for these injuries. We believe that the treatment plan should be tailored for each case depending mainly on the femoral neck fracture displacement and the surgeon’s’ experience with the available implants.

Mohapatra et al. reported satisfactory results using both single versus dual constructs in 18 cases of ipsilateral femoral neck and shaft fractures [1]. Similarly, Wei et al. reported no significant difference in fractures reduction or complications in 22 patients treated with single versus dual implants [12].

The complexity of ipsilateral femoral neck and shaft fractures mandates careful preoperative planning on an individual basis. Both fractures should be treated with an implant(s) that optimizes fracture healing while simultaneously prioritizing the femoral neck fracture to avoid avascular necrosis [1, 4, 6, 13]. In one of the largest series on the topic, Oh et al. reported on 74 cases of ipsilateral femoral neck and shaft fractures. The rate of avascular necrosis was 6.8% with higher risk with displaced femoral neck fractures. The authors also reported a high rate of femoral shaft nonunion of 20% [14]. Although Hung et al. [15] reported that the order of fixation of the fractures may not be important, others gave priority to fixation of the femoral neck fractures first especially if displaced. Some authors even support fixing the femoral shaft fracture first as it will aid in the fixation of the femoral neck [16–18]. Recently, The American Academy of Orthopedic Surgeons published an article in which they described a preferred approach to guide surgeons in fixing ipsilateral femoral neck and shaft fractures [16]. If the femoral neck fracture was displaced, it is preferred to use two implants to fix the fractures starting with fixing the neck of femur. Regarding femoral shaft fracture, after fixation, surgeons should take radiographs of the contralateral side to evaluate for length, alignment and malreduction. If there is no obvious neck of femur fracture preoperatively, an intraoperative fluoroscopic examination should be done to assess for fracture. Prophylactic measures should be taken if there is no femur neck fracture.

Multiple surgical fixation techniques are used based on patients’ factors, fracture characteristics and surgeon preference, with controversial clinical outcomes reported in the literature. Treatment options include single constructs (e.g. CMN, long sliding hip screw) and dual constructs (e.g. retrograde nail with sliding hip screw, proximal femoral locking plate or cannulated screws) [16]. While each has its own merits and demerits, the goal of any treatment plan should be an anatomic reduction of neck fracture and stable fixation of both fractures so patients can be early mobilized [19].

The CMN is a one implant option used to fix both fractures. The advantages of CMN include using a single incision to fix both fractures and saving the bony stock for the insertion of proximal screws for hip fractures [7]. In addition, the infection rate and blood loss can be reduced with the closed reduction technique. The CMN is known to be a load-sharing device that allows early rehabilitation postoperatively [7]. This construct, however, is technically demanding and associated with high malreduction rates [11, 20]. Bedi et al. found a statistically significant higher rate of malreduction of either femoral neck or shaft fracture when CMN is used to address both fractures simultaneously. They advised using two implants rather than one to avoid malreduction [11]. The most important finding of the current study is that there was no statistically significant difference between the two groups regarding fracture union, the accuracy of reduction and implant failure.

### Limitations

This study is limited by its retrospective nature, single-site perspective, small cohort due to the rarity of the injury and the short-term follow-up period. Although we assessed the radiological outcomes postoperatively, the functional outcomes could not be assessed due to incomplete data available for analysis. In addition, our results may not be generalized to other practice settings due to the different surgeons’ preferences and various surgical implants available.

## Conclusions

Ipsilateral femoral neck and shaft fractures are rare high-energy trauma that required a high level of suspicion and planned early management. Early surgical fixation of both fractures was associated with good outcome results. Single versus dual implant fixation was not found to significantly affect the radiological outcomes or complications. High-level multicenter studies with long-term follow-up would be needed to further investigate the optimal management of such injuries.

## Supplementary Information

Below is the link to the electronic supplementary material.

Supplementary file1 (DOC 104 KB)

## Data Availability

Data available on request from the authors.
