# Correction to: The surgical outcomes of fixing ipsilateral femoral neck and shaft fractures: single versus double implants fixation

**DOI:** 10.1007/s00590-022-03411-y

**Published:** 2022-10-15

**Authors:** Yahya Alborno, Abdullah M. Abunimer, Yousef Abuodeh, Motasem Salameh, Hammam Kayali, Ghalib Ahmed

**Affiliations:** 1 Department of Orthopedic Surgery, Hamad General Hospital, Doha, Qatar; 2 Miller School of Medicine, University of Miami Hospital-Jackson Memorial Hospital, Miami, USA; 3 Orthopedic Surgery Department, Brown University, Providence, RI USA

## Correction to: European Journal of Orthopaedic Surgery & Traumatology 10.1007/s00590-022-03312-0

The original version of this article unfortunately contained a mistake. The author name Abdullah M. Abunimer was incorrectly written as Abdullah Abunimer.

